# Atlas of Skin Diseases

**Published:** 1877-10

**Authors:** 


*Atlas of Skin Diseases.* Part II. Acne rosacea, Ichthyosis, Tinea vesicolor, Sycosis non-parasitica. By Louis A. Duhring, M. D. Philadelphia: J. B. Lippincott & Co., 1877.

It is now about a year since we had occasion to notice Dr. Duhring's Atlas. We predicted at the time that the subsequent numbers would fully bear out the promise of excellence indicated by the first part; nor are we disappointed, on the contrary we are again surprised at the exactness with which the artist under Dr. Duhring's skillful direction has succeeded in counterfeiting nature. Some little difficulty has been experienced in printing and hence the delay in the appearance of Part II, but we are informed that subsequent numbers will appear promptly. The delay can be easily overlooked when we consider the care that has been expended in producing the plates in this number.

Plate I. Acne rosacea represents a severe form of that affection, more so in fact than is usually met with but it is, nevertheless, true to life. The enlarged capillaries and generally congested condition of the skin are well represented. The appearance of the comedones is well shown. The letter press accompanying the plate is concise and excellently clear, the treatment recommended is such as will in most cases meet with success. It will of course have to be modified to suit individual cases.

Plate II. Represents most excellently Ichthyosis, the appearance of the skin is excellently drawn and especially fine representation is made of the thickened and hardened appearance so frequently noticed at the elbows and knees.

In Plate III, Tinea vesicolor or what is frequently called liver spots by non-professional people is represented. The author in the letter press warns the readers against confounding it with liver spots or chloasma and with certain of the syphilitic eruptions. The mistake cannot be made if the physician will call his microscope to his aid for the mycelium and spores are easily discoverable under a power of three hundred diameters.

Plate IV is especially interesting for the reason that it represents a disease, Sycosis non-parasitica, which is so often treated as if due to a parasite and consequently the patient's troubles are but continued and frequently augmented. By carefully studying the text and observing this plate the reader need not make this mistake.

Dr. Duhring's attempt to make an American Atlas of Skin Diseases has certainly been successful as far as excellence and truthfulness of work is concerned, we hope he will be equally successful in receiving the substantial encouragement of the profession. B.

